# Antidepressant effect of ketamine unrelated to dissociation: Results from an exploratory mediation analysis of the KET01-02 study

**DOI:** 10.1192/j.eurpsy.2025.471

**Published:** 2025-08-26

**Authors:** L. Arvastson, E. Papanastasiou, K. Schmid, A. Damyanova, A. Glas, C. Strote, C. Eulenburg, D. Gehrlach, K. Maiboe, H. Eriksson

**Affiliations:** 1 HMNC Brain Health, Munich, Germany; 2 Develco Pharma, Pratteln, Switzerland

## Abstract

**Introduction:**

Current ketamine-based therapies for treatment-resistant depression (TRD) can induce dissociative symptoms. A novel oral prolonged-release ketamine formulation (KET01) results in a lower and delayed peak concentration of ketamine, and a higher concentration of the metabolites norketamine and hydroxynorketamine than after intravenous administration. KET01 has limited dissociative properties, compared to other ketamine formulations.

**Objectives:**

To explore the relation between dissociative and antidepressant effects of KET01.

**Methods:**

KET01-02 (EudraCT 2021-004927-34) was a randomized, double-blind phase 2 trial in outpatients with TRD comparing adjunct 120 mg (n=42) or 240 mg (n=40) oral KET01 once-daily for 3 weeks to placebo (PBO, n=40). The primary endpoint was change from baseline on the Montgomery-Åsberg Depression Rating Scale (MADRS) total score on day 21. Dissociation was assessed using the Clinician-Administered Dissociative States Scale (CADSS).

The association between CADSS scores at 7 hours after first dosing and MADRS scores on day 4 was investigated with a statistical mediation analysis. The 7-hour timepoint was selected since it coincides with the average Tmax (time-to-peak) when the highest dissociation is expected. Depression scores at the first subsequent visit (on day 4) were selected for the analysis. It was also the time point where change in MADRS score from baseline differentiated the most between KET01 and placebo with a difference of 4.32 (p=0.006) to the benefit of KET01 – based on the model used in the mediation analysis.

**Results:**

The antidepressant effect of KET01 that was mediated through dissociation was estimated to the negligible -1.28% (CI: (-28%) – (+11%)).

**Conclusions:**

The antidepressant effect of KET01 was achieved with minimal to no dissociation and with no significant mediation through dissociation. Our findings challenge the commonly held clinical view that some degree of dissociation is necessary to guarantee ketamine’s antidepressant effect. Instead, it appears that dissociative symptoms are merely adverse events associated with certain formulations of ketamine.

**Disclosure of Interest:**

L. Arvastson Consultant of: HMNC Brain Health, E. Papanastasiou Employee of: HMNC Brain Health, K. Schmid Employee of: Develco Pharma, A. Damyanova Employee of: HMNC Brain Health, A. Glas Employee of: HMNC Brain Health, C. Strote Employee of: HMNC Brain Health, C. Eulenburg Consultant of: HMNC Brain Health, D. Gehrlach Employee of: HMNC Brain Health, K. Maiboe Consultant of: HMNC Brain Health, H. Eriksson Employee of: HMNC Brain Health

